# Diverticulitis Is Associated with Increased Risk of Colon Cancer—A Nationwide Register-Based Cohort Study

**DOI:** 10.3390/jcm13092503

**Published:** 2024-04-24

**Authors:** Laura Quitzau Mortensen, Kristoffer Andresen, Lau Thygesen, Hans-Christian Pommergaard, Jacob Rosenberg

**Affiliations:** 1 Center for Perioperative Optimization, Department of Surgery, Herlev Hospital, University of Copenhagen, 2730 Herlev, Denmark; lauraquitzau@outlook.com (L.Q.M.);; 2 Department of Radiology, Centre for Functional and Diagnostic Imaging and Research, Amager and Hvidovre Hospital, 2650 Hvidovre, Denmark; 3 National Institute of Public Health, University of Southern Denmark, 1455 Copenhagen, Denmark; 4 Hepatic Malignancy Surgical Research Unit (HEPSURU), Department of Surgery and Transplantation, Rigshospitalet, 2100 Copenhagen, Denmark; 5 Department of Clinical Medicine, University of Copenhagen, 2200 Copenhagen, Denmark

**Keywords:** diverticular disease, survival analysis, colorectal cancer, colonoscopy

## Abstract

**Background:** An association between diverticulitis and colon cancer has been proposed. The evidence is conflicting, and the guidelines differ regarding recommended follow-up with colonoscopy after an episode of diverticulitis. To guide regimes for follow-up, this study aimed to investigate if patients with diverticulitis have an increased risk of colon cancer. **Methods:** This study is reported according to the RECORD statement. We performed a cohort study with linked data from nationwide Danish registers. The inclusion period was 1997–2009, and the complete study period was 1995–2013. The primary outcome was the risk of developing colon cancer estimated using a Cox regression analysis with time-varying covariates. We performed a sensitivity analysis on a cohort of people with prior colonoscopies, comparing the risk of colon cancer between the diverticulitis group and the control group. **Results:** We included 29,173 adult males and females with diverticulitis and 145,865 controls matched for sex and age. The incidence proportion of colon cancer was 2.1% (95% confidence interval (CI) 1.9–2.3) in the diverticulitis group and 1.5% (95% CI 1.4–1.5) in the matched control group (hazard ratio 1.6; 95% CI 1.5–1.8). The risk of having a colon cancer diagnosis was significantly increased in the first six months after inclusion (hazard ratio 1.7; 95% CI 1.5–1.8), and hereafter there was a lower risk in the diverticulitis group compared with controls (hazard ratio 0.8; 95% CI 0.7–0.9). This protective effect lasted eight years. The increased risk of colon cancer during the first six months after diverticulitis was also found in the cohort with prior colonoscopies. **Conclusions:** The risk of a colon cancer diagnosis was significantly increased for patients with diverticulitis 0–6 months after the diagnosis of diverticulitis. Hereafter, we found a protective effect of diverticulitis until eight years later, possibly due to a screening effect. We recommend a follow-up colonoscopy after the first diagnosis of diverticulitis.

## 1. Introduction

Diverticulitis of the colon is a common disease. Approximately 60% of those above the age of 60 have diverticulosis coli [1] and approximately 4% develop diverticulitis [2]. Also, the incidence of diverticulitis is increasing in the younger population [3]. The pathophysiology of diverticulitis is still unclear. However, new research indicates that mucosal inflammation and a change in the gut microbiome might be involved [4]. Colon cancer is the fourth most common cancer in the world with a worldwide incidence of 1,096,601 cases each year [5]. It has been proposed that inflammation may play a role in the association between diverticulitis and colon cancer [6]. Thus, inflammatory bowel disease in the colon has been found to increase the risk of developing colon cancer [7]. In addition, specific inflammatory biomarkers have been associated with both colorectal neoplasia and diverticulitis [8].

There are conflicting results in the literature regarding the association between diverticulitis and colon cancer [9,10,11]. In a previous study, we found that patients with diverticulitis had an increased risk of colon cancer [10]. However, due to the design of the study, it was not possible to deduce a temporal relationship. Other studies point to the risk being increased in the first year after diverticulitis [12,13,14,15]. However, none of these studies have a long-term follow-up for examining the relationship between the two diseases.

This study aimed to investigate a temporal relationship between diverticulitis and the subsequent development of colon cancer.

## 2. Materials and Methods

This study is reported according to the ‘reporting of studies conducted using observational routinely-collected data’ (RECORD) statement and, where applicable, an extension of the ‘strengthening the reporting of observational studies in epidemiology’ (STROBE) guideline [16]. Permission from the Danish Data Protection Agency was granted [HEH-2013-056]. In Denmark, it is not mandatory to obtain ethical or patient approval for register-based studies.

We performed a register-based cohort study with information from nationwide Danish registers on the entire Danish population from 1995–2013. The inclusion period was 1997–2009. The diverticulitis cohort was identified, and the inclusion date was defined as the date of the first admission with diverticulitis. The diverticulitis group was matched in a 1:5 ratio for sex and year of birth with controls from the general population who had no primary or secondary diagnosis of diverticulitis as an inpatient or outpatient before or at the inclusion date. The inclusion dates of the controls corresponded to the inclusion dates of their matched participants with diverticulitis. Therefore, the two groups were age-matched. The matching was performed using a reworked version of a previously published program [17]. We included a look-back period from 1995–1996, allowing for exclusion based on specific diseases and surgical procedures for at least two years back in time. The follow-up lasted until the end of 2013, giving a minimum follow-up of at least four years for all participants.

We included male and female adults of 18 years and above. Only those who were Danish residents since the 1st of January 1995 and until their inclusion date were eligible for inclusion. We identified the group with diverticulitis as having been admitted to the hospital with a primary diagnosis of diverticulitis during the inclusion period (see Supplementary Material Table S1, which provides a complete list of diagnostic codes used to identify the cohort). Participants were excluded if they had been admitted with a primary diagnosis of diverticulitis in the look-back period or if they had had a total colectomy or colon cancer before the inclusion date (see Supplementary Material Table S1 for a complete list of diagnostic and procedural codes).

The event in this study was a diagnosis of colon cancer in the Danish Cancer Registry (see Supplementary Material Table S1, which contains a complete list of diagnostic codes). The participants were censored upon the end of the study (31 December 2013), emigration, death, or total colectomy (see Supplementary Material Table S1). If a person from the control group was admitted with diverticulitis as the primary diagnosis, then it resulted in censoring at the date of diverticulitis. If the admission with diverticulitis was during the inclusion period, then the person would have a new inclusion date in the diverticulitis group. These participants therefore appear as two records in the data set. For the group with diverticulitis, a resection of the colon in relation to a diagnosis of diverticulitis also resulted in censoring with the assumption that the removed colon segment was the one with diverticulitis. However, if the same person had a registration of colon cancer up to two months after the resection, then the patient was registered with an event instead. We did this to account for a possible delay in the pathology diagnosis of colon cancer.

The primary outcome of this study was the incidence of colon cancer. Secondary outcomes were mortality, cancer stage (see Supplementary Material Table S2, which provides information on the cancer stages), and any difference herein between the two groups. We performed a subgroup analysis on the group with diverticulitis to compare the incidence of colon cancer between patients with complicated and with uncomplicated diverticulitis (see Supplementary Material Table S1 for definitions). Surgery, in the form of resection of the colon, was accounted for in the analysis as an additional indicator of the severity of disease (see Supplementary Material Table S1 for procedure codes). Another secondary outcome was a sensitivity analysis where the primary outcome was examined in the cohort that had not been censored or that had an event from the inclusion date until six months after. This group was examined from six months after the inclusion date and until censoring or an event. A second sensitivity analysis was performed, examining the primary outcome in the cohort who had had a colonoscopy within two years prior to their inclusion and who were therefore considered to enter the study period with a clean colon.

The study was conducted with data from the Danish National Patient Register, the Danish Register of Causes of Death, the Danish Cancer Registry, and the Danish National Health Service Register. The registers are linkable with the civil registration number, which is a unique 10-digit number given at birth or upon residency [18]. Data were pseudonymized before we received them. The Danish National Patient Register was started in 1977, and since 1995 all somatic and psychiatric admissions have been registered including both in- and outpatient contacts [19]. The register contains information from both public and private hospitals, and reporting to the register has been compulsory since 2003 [19]. Since 1994, the International Classification of Diseases, Tenth Revision, (ICD-10) has been used [19]. To classify surgeries and procedures, the Danish Classification of Surgical Procedures and Therapies, Third Edition, was used from 1989 to 1995, and the Nordic Medico-Statistical Committee Classification of Surgical Procedures has been used since 1996 and to date [19].

The Danish Register of Causes of Death has existed in electronic form since 1970 [20]. The register contains information on the causes of all deaths registered in Denmark [20]. The Danish Cancer Registry has existed since 1943 and registration has been mandatory since 1987 [21]. The register contains information on all new cancers in Denmark, including the morphology and topography. The register is cross-referenced with the Danish National Patient Register, the Danish Pathology Registry [22], and the Danish Register of Causes of Death to identify any cancers missing from the register [21]. Since 1978, the ICD-10 has been used to classify the diagnosis, and the International Classification of Diseases for Oncology, Third Edition, (ICD-O-3) to characterize the morphology and topography [21] (see Supplementary Material Table S2). The Danish National Health Service Register has existed since 1990 and contains information on every contact with private clinics and general practitioners [23]. Registrations are based on invoices from the clinics to the state, and the coverage is therefore assumed to be high [23]. For this study, information on colonoscopies performed in private surgery clinics was derived from this register.

Unadjusted analyses examined categorical variables using the chi-squared test, log-rank test, and Cox regression. Adjusted analyses were performed using the Cox regression, using the ‘proc phreg’-statement in the SAS© software, version 9.4, and adjusting for age at inclusion, as well as the potential confounders Crohn’s disease, ulcerative colitis, and diabetes [24] (see Supplementary Material Table S1 for diagnostic codes). We also included in the Cox regression a time-dependent covariate based on colonoscopies (see Supplementary Material Table S1 for procedure codes). The participants were marked as having a clean colon from the registration of a colonoscopy and until two years later. If the participants had another colonoscopy within the two-year period, then the period was extended by two years from the latest colonoscopy date. If colon cancer was registered two months or less after a colonoscopy, then the cancer was assumed to have been found at the colonoscopy, and the delay in diagnosis to be due to processing of the pathologic tissue, and these patients would therefore not be registered as having a clean colon. *p*-values less than 5% were considered significant.

The output, code, and data analysis for this paper was generated using SAS© software, version 9.4. Kaplan-Meier plots were generated using SPSS statistics, version 24 [25].

## 3. Results

**Figure 1:**
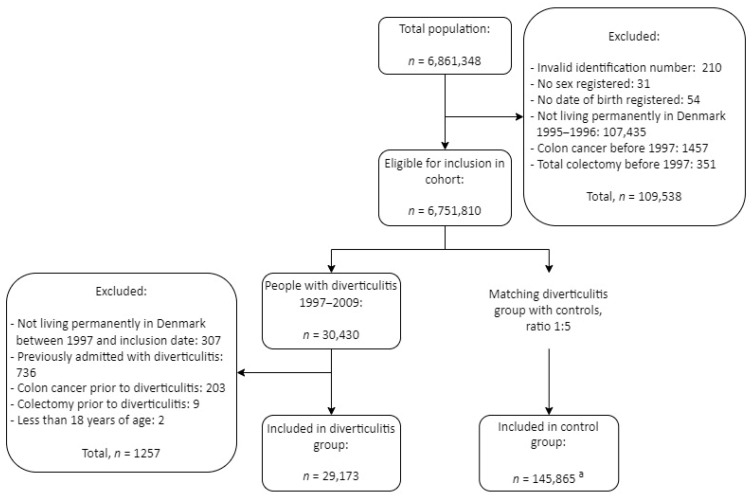
Selection process. “Total population” represents every unique personal identifier registered in the period of 1995–2013 in Denmark. ^a^ 1099 controls developed diverticulitis and therefore exist in both the diverticulitis and control group.

We included 175,038 patients in this study, i.e., 29,173 patients with diverticulitis, who were matched with five controls each, resulting in the inclusion of 145,865 controls without diverticulitis. There were 1099 from the control group who developed diverticulitis after inclusion and therefore existed in both groups. See Figure 1 for a flowchart of the inclusion process.

The two groups were similar regarding age, sex, and follow-up time, but there was a greater prevalence of Crohn’s disease, diabetes, and ulcerative colitis in the diverticulitis group (Table 1).

**Figure 2:**
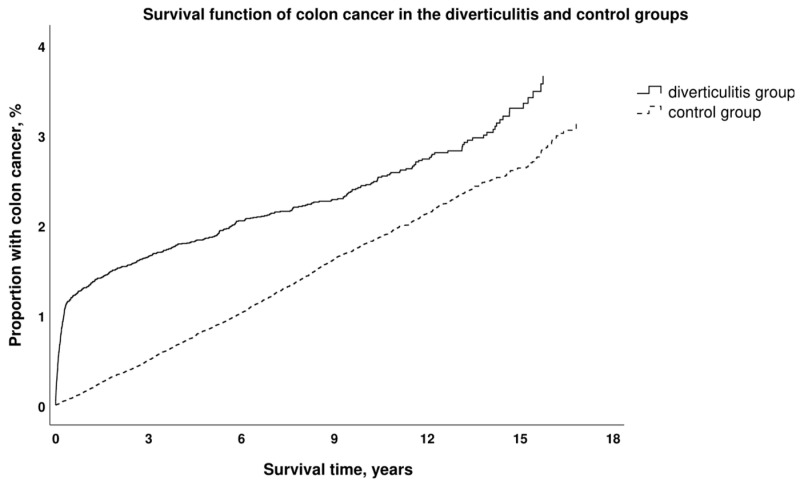
Kaplan–Meier curve shows the cumulative hazard of colon cancer incidence with time between the group with diverticulitis and the control group without diverticulitis.

The incidences of colon cancer in the two groups are depicted in Table 2. The unadjusted Cox regression gave a hazard ratio (HR) of colon cancer in the diverticulitis group compared with the control group of 1.6 (95% confidence interval (CI) 1.5–1.8). The Cox regression adjusted for covariates gave an HR of 1.7 (95% CI 1.5–1.8). The survival function for the unadjusted model can be seen in Figure 2.

The sensitivity analysis, which examined the cohort from six months after inclusion, found a decreased risk of colon cancer for the diverticulitis group compared with the controls (HR 0.8; 95% CI 0.7–0.9). The survival function is portrayed in Supplementary Material Figure S1. Eight years after inclusion, the risk of colon cancer was equal between the remaining participants in the two groups (HR 0.8; 95% CI 0.6–1.01). The second sensitivity analysis, which examined the cohort that had a clean colon at inclusion, found an increased risk for the group with diverticulitis within the first six months after inclusion (HR 3.9; 95% CI 1.2–12.7), but no significant difference overall (HR 0.8; 95% CI 0.5–1.3). The characteristics of the cohorts used in the sensitivity analyses remained comparable (Table 3).

The first six months after the diagnosis of diverticulitis, the patients had an increased risk of stage III colon cancer and a decreased risk of stage I cancer (Table 4). From six months, the group with diverticulitis had a decreased risk of stage IV colon cancer when compared with the group without diverticulitis (Table 4). There were missing data on cancer stage in both the diverticulitis and control groups (12.9% and 17.6%, respectively; Table 4). Mortality was increased in the group with diverticulitis (Table 2).

The subgroup analysis in the diverticulitis group showed an even distribution of colon cancer events in the groups with complicated and uncomplicated diverticulitis; see Table 5. However, when analyzed with a Cox regression adjusted for age, Crohn’s disease, ulcerative colitis, diabetes, and a time-dependent variable for a clean colon, we found an increased risk of colon cancer in those with complicated diverticulitis compared with uncomplicated diverticulitis (HR 1.6; 95% CI 1.2–2.0). After adjustment for surgery in the model, the HR decreased to 1.4 (95% CI 1.01–1.7).

## 4. Discussion

We found an increased risk of developing colon cancer for patients with diverticulitis compared with controls without diverticulitis. The increase was limited to the first six months after the diagnosis of diverticulitis. After the first six months, there was a decreased risk of colon cancer in the group with diverticulitis until eight years after the diagnosis. The increased risk during the first six months was also identified in the sensitivity analysis of the cohort with colonoscopy up to two years before inclusion. The subgroup analysis showed an increased risk of colon cancer for patients with complicated diverticulitis compared with patients with uncomplicated diverticulitis.

This study was performed using data linked at the individual level from nationwide and comprehensive Danish registers [19,20,21,22,23]. The size of the cohort provided the necessary power to investigate colon cancer, which is a rare event. We were able to have at least two years of look-back and the possibility of four years of follow-up for everyone included in the study. This allowed the inclusion of a homogenous group with no prior colon cancer or diverticulitis and a long follow-up time to identify colon cancer. However, our study was, as any other register-based study, dependent on clinicians registering diagnoses and procedures correctly and thoroughly, with a risk of misclassification bias and missing data [26]. It was not possible to check whether the data were correctly coded. Furthermore, the classification codes for diverticulosis and diverticulitis have a high overall positive predicted value, but are not completely reliable for differentiating accurately between diverticulosis and diverticulitis or the severity of diverticulitis [27]. In order to accommodate this, we only included patients with a primary and inpatient diagnosis of diverticulitis. Most likely, we did not include any patients with just diverticulosis. However, it is possible that we included some with symptomatic uncomplicated diverticular disease (SUDD). There are no data on the admission rate of SUDD in the literature. However, the symptoms are somewhat equal to those of irritable bowel syndrome [28], and one may therefore hypothesize that the majority of those with SUDD are handled in outpatient settings. Since the diagnosis codes have a lower validity in distinguishing between the severity of disease, we used additional codes of abscess and procedures, i.e., abscess drainage and peritoneal lavage, in order to better identify complicated cases of diverticulitis. The risk factors for colon cancer and diverticulitis are overlapping, and due to the contents of the registers, we were not able to consider this confounding risk in our study. Specifically, smoking, lifestyle, body mass index, and diet are risk factors for both diseases, and data on these parameters are not available in the national registers [29,30,31,32]. The time of diagnosis may vary by a few months, for example, when pathology is needed to confirm the diagnosis. We attempted to account for this delay when marking for a clean colon after a colonoscopy. Approximately 16% of those with a colon cancer diagnosis had missing information regarding cancer topography. This could lead to information bias. However, there was no marked difference between the two groups.

It is possible that the increased incidence of colon cancer that we found within the first six months was due to a misdiagnosis of diverticulitis at the primary admission and corrected to the diagnosis of colon cancer during the colonoscopy a few months later. However, a high accuracy has been established in detecting diverticulitis with computed tomography (CT) [33,34,35], and a large Norwegian study also found an increased risk of colorectal cancer in patients with CT-verified diverticulitis [14]. It therefore seems plausible that the diverticulitis and colon cancer found within six months of each other in our study existed concurrently at the time of diagnosis of diverticulitis.

We found an increased mortality in the diverticulitis group, which is similar to other studies [36,37] that have found a worse prognosis for patients with diverticulitis and colon cancer than for patients without diverticulitis. In the diverticulitis group, we found an increased risk of stage III colon cancer during the first six months and a decreased risk of stage I colon cancer. Our results are consistent with another study that examined patients with conservatively treated and CT-verified diverticulitis, which found an increased risk of higher stages of colon cancer [36]. A study on the Danish colorectal cancer screening program, which begun in 2014, found that invited compared with not-yet-invited participants had an increased risk of lower cancer stages [38], and the diverticulitis group in our study consequently does not resemble the screening population. Using a sensitivity analysis, we examined the participants with a colonoscopy two years or less before the inclusion date and who were therefore considered to have a clean colon at inclusion. We performed this analysis to explore if the increased risk in the diverticulitis group was a result of endoscopic examination after the diverticulitis, resulting in a screening effect. This analysis showed no increased overall risk for the patients with diverticulitis. However, it did find a significantly increased risk of colon cancer during the first six months, thereby contradicting the theory of increased findings of cancer due to a screening effect. This result may suggest a relationship between diverticulitis and colon cancer, an incorrect first diagnosis, an unrecognized common risk factor, or a common risk factor that could not be adjusted for in our analysis. This result also challenges the paradigm of sustaining from a colonoscopy following diverticulitis, if there is a recent colonoscopy [39,40]. A recent retrospective study had findings contradictive to ours [11]. They found that patients with diverticulitis and a colonoscopy up to five years prior did not have an increased risk of colon cancer. The cohort, however, was small, with only 124 patients with diverticulitis and a previous colonoscopy.

We found a decreased risk of developing colon cancer in the group with diverticulitis from six months after the diverticulitis diagnosis and until eight years after. We believe this to be due to a screening effect from the follow-up colonoscopy. Previous studies have found that 14–31% had adenomas upon colonoscopy after an episode of diverticulitis [41,42,43,44]. In a recently published systematic review, we also found that there was no increased long-term risk of colon cancer from six months after an episode of diverticulitis [45]. We found an increased risk of colon cancer in patients with complicated diverticulitis compared with uncomplicated diverticulitis in the adjusted analysis. Our results are aligned with the conclusions of two recent meta-analyses [42,46]. In our analysis, the difference in risk was only merely significant when adjusting for surgery. This may indicate that the severity of symptoms plays an important role in assessing patients’ risk. This was also the conclusion of a study that found an increased risk of colorectal cancer for patients with uncomplicated diverticulitis and alarm symptoms [36].

The international guidelines on patient management after an episode of diverticulitis differ regarding the follow-up colonoscopy. The vast majority of guidelines recommend a follow-up colonoscopy after an episode of diverticulitis, either in all patients, those with complicated diverticulitis, with persisting symptoms, or without a recent colonoscopy [39,40,47,48,49,50,51]. In Denmark, the national guideline recommends a colonoscopy when the acute phase of diverticulitis has passed, which is usually about six to eight weeks after the first admission [50]. Our study was performed using data from comprehensive, national, Danish registers, linked at an individual level and including adults with a hospital admission for diverticulitis. The applicability of our results is high for countries with similar living standards and a similar composition of age and ethnicity to the Danish population. Our results, however, are probably not applicable to countries with a very different incidence or localization of diverticulitis, i.e., African and Asian countries [52,53]. The results of this study support the continued practice of performing a colonoscopy after an episode of diverticulitis. However, specific recommendations regarding the optimal timing or frequency of colonoscopy examinations cannot be made based on these results.

## 5. Conclusions

We found a significantly increased risk of colon cancer in patients with diverticulitis compared with people without diverticulitis. The increase was apparent up to six months after the diagnosis of diverticulitis followed by a lowered risk from six months after a diagnosis of diverticulitis to approximately eight years after. Due to the register-based study design, the study limitation included a risk of misclassification bias. These results support the recommendation of a follow-up colonoscopy within the first six months after the first diagnosis of diverticulitis. Future studies should ideally explore this topic in a prospective setting.

## Figures and Tables

**Table 1:** Characteristics of the cohort. SD = standard deviation.

	Diverticulitis Group *n* = 29,173	Control Group *n* = 145,865
Age at inclusion, years, mean (SD)	67.4 (14.6)	67.4 (14.6)
Female, *n* (%)	17,669 (61)	88,345 (61)
Follow-up, years, mean (SD)	7.2 (4.7)	8.1 (4.3)
Crohn’s disease, *n* (%)	355 (1)	445 (0.3)
Diabetes, *n* (%)	3817 (13)	13,080 (9)
Ulcerative colitis, *n* (%)	538 (2)	942 (1)

**Table 2:** Cancer and mortality. * Unadjusted Cox regression. † Adjusted Cox regression. CI = confidence interval.

	Diverticulitis Group *n* = 29,173	Control Group *n* = 145,865	
Colon cancer, *n* (%)			Hazard ratio (95% CI)
In total	611 (2)	2101 (1)	1.6 (1.5–1.8) *
			1.7 (1.5–1.8) †
From 6 months and forward	288 (1)	1995 (1.4)	0.8 (0.7–0.9) *
			0.8 (0.7–0.9) †
Mortality, *n* (%)			Log-rank test
In total	12,639 (43)	53,885 (37)	*p* < 0.001
From 6 months and forward	10,938 (38)	51,468 (35)	*p* < 0.001

**Table 3:** Characteristics of the cohorts used in the two sensitivity analyses. The first sensitivity analysis explored the risk of colon cancer in the diverticulitis group and control group from 6 months to 8 years after inclusion. The second sensitivity analysis explored the cohort with colonoscopy until two years before inclusion. SD = standard deviation.

**First Sensitivity Analysis, Risk from 6 Months after Inclusion.**		
From six months after inclusion	Diverticulitis group, *n* = 25,925	Control group, *n* = 143,190
Age at inclusion, years, mean (SD)	67 (15)	67 (15)
Female, *n* (%)	15,761 (61)	86,714 (61)
Colon cancer, *n* (%)	288 (1.1)	1995 (1.4)
From eight years after inclusion	Diverticulitis group, *n* = 12,092	Control group, *n* = 68,508
Age at inclusion, years, mean (SD)	62 (13)	63 (13)
Female, *n* (%)	7390 (61)	41,737 (61)
Colon cancer, *n* (%)	69 (0.6)	496 (0.7)
**Second Sensitivity Analysis, Colonoscopy Prior to Inclusion**		
	Diverticulitis group, *n* = 2549	Control group, *n* = 2334
Age at inclusion, years, mean (SD)	68 (13)	70 (12)
Female, *n* (%)	1529 (60)	1369 (59)
Colon cancer, *n* (%)		
In total	25 (1)	34 (1.5)
First six months	10 (0.4)	4 (0.2)

**Table 4:** Colon cancer stages presented for those detected from inclusion date to six months after and from six months after inclusion to end of study. Stage X stems from the old classification used in Denmark until 2003 and represents both stage I and stage II colon cancers; please refer to Supplementary Material Table S2. T = tumor staging, N = node staging, M = metastasis staging, and CI = confidence interval. * *p*-value less than 5%.

Cohort with Events during the First Six Months after Inclusion, *n* = 428			
Colon Cancer Stage	Diverticulitis Group, *n* (%)	Control Group, *n* (%)	Risk Ratio (95% CI)
I	9 (3)	9 (9)	0.3 (0.1–0.8) *
II	105 (33)	27 (26)	1.3 (0.9–1.8)
III	102 (32)	22 (21)	1.5 (1.01–2.3) *
IV	58 (18)	20 (19)	0.9 (0.6–1.5)
X	15 (5)	10 (10)	0.5 (0.2–1.1)
Missing info on T, N, or M	34 (11)	17 (16)	0.7 (0.4–1.1)
Total	323 (100)	105 (100)	
**Cohort with Events from Six Months after Inclusion to End of Study, *n* = 2284**			
I	25 (9)	148 (7)	1.2 (0.8–1.8)
II	84 (29)	527 (26)	1.1 (0.9–1.3)
III	78 (27)	485 (24)	1.1 (0.9–1.4)
IV	44 (15)	432 (22)	0.7 (0.5–0.9) *
X	12 (4)	51 (3)	1.6 (0.9–3.0)
Missing info on T, N, or M	45 (16)	353 (18)	0.9 (0.7–1.2)
Total	288 (100)	1996 (100)	

**Table 5:** Distribution of colon cancer in the exposed cohort based on severity of diverticulitis.

	Uncomplicated Diverticulitis Group, *n* (%)	Complicated Diverticulitis Group, *n* (%)
Colon cancer	531 (2)	80 (2)
No colon cancer	24,980 (98)	3582 (98)
Total	25,511	3662

## Data Availability

The data used in our study are from national Danish registers. Access is granted via the Danish Health Data Authorities. The requirements for obtaining access are located online at www.sundhedsdatastyrelsen.dk (in Danish), latest accessed on 24 April 2024.

## Supplementary Materials

The following supporting information can be downloaded at: https://www.mdpi.com/article/10.3390/jcm13092503/s1, Table S1. Diagnostic codes used to identify the diverticulitis cohort, colon cancer events, and confounders. Table S2. Cancer classifications used in The Danish Cancer Registry (DCR) and overview of cancer stages. Figure S1. Kaplan Meier Curve showing the cumulative hazard of colon cancer incidence with time between the group with diverticulitis and the control group without diverticulitis. References [54,55,56] are cited in the Supplementary Materials.
