# Supplementary material for: Diverticulitis Is Associated with Increased Risk of Colon Cancer—A Nationwide Register-Based Cohort Study
Source: J Clin Med. 2024 Apr 24;13(9):2503. doi: 10.3390/jcm13092503 (PMC11084441; doi:10.3390/jcm13092503)

Supplementary material 1

Table S1. Diagnostic codes used to identify the diverticulitis cohort, colon cancer events, and confounders. NOMESCO = Nordic Medico-Statistical Committee Classification of Surgical Procedures.[54] ICD-10 = International Classification of Diseases and Related Health Problems 10th Revision.[55] DNHSR = The Danish National Health Service Register.[23] DNPR = The Danish National Patient Register.[19]

Classification system	Disease, operation, or procedure	Codes	Source register
ICD-10	Diverticulitis:		
	– All diagnoses	K572, K572A-C, K573, K573A, K573B.	DNPR
	– Uncomplicated diverticulitis diagnoses	K573, K573A-B.	DNPR
	– Complicated diverticulitis diagnoses	1) K572, K572A-C.<br>or<br>2) K573, K573A-B. and additional diagnosis for abscess: K630, K650A, K650C-J. or procedure code (NOMESCO) for abscess drainage or peritoneal lavage: KJAJ, KJAJ00, KTJA40, KJAA00A, KJAK00, KJAK01, KJAK, KJAK03, KJAK04.	DNPR

	Colon cancer	C18 including all subcodes except C188A and C189X.	The Danish Cancer Registry[21]
	Crohn's disease	K50, K501, K501D, K508, K508D, K508A, K509, M074, O996A.	DNPR
	Ulcerative colitis	K51, K515, K519, K518, M092, O996B, M075.	DNPR
	Diabetes	E10 including all subcodes, E11 including all subcodes, E13, E134, E136, E137, E138, E14 including all subcodes, N083, ALAL22	DNPR
NOME SCO	Total colectomy	KJFH, KJFH00-01, KJFH10-11, KJFH20-21, KJFH30-31, KJFH33, KJFH40, KJFH96.	DNPR
	Colon resection	KJFB, KJFB20-21, KJFB30-31, KJFB33-34, KJFB40-41, KJFB43-44, KJFB46-47, KJFB50-51, KJFB60-61, KJFB63-64.	DNPR
	Colonoscopy	KUJF32, KUJF35,<br>Fee number used in DNHSR: 2114	DNPR and DNHSR

Supplementary material 2

Table S2. Cancer classifications used in The Danish Cancer Registry (DCR) and overview of cancer stages. * Classification system used in DCR 1943–2003. † Classification system used in DCR 2004 and to date.[21] ‡ Colon cancer stages based on the cancer staging manual by American Joint Committee on Cancer.[56]

Classification (years of use)	Content of classification	Cancer stage ‡
DUKE (1943–2003) *	· DUKE A: Confined to the inner lining of the bowel<br>· DUKE B: Tumor grown through muscle layer of the bowel<br>· DUKE C: Spread to at least one lymph node in proximity to the cancer<br>· DUKE D: Distant metastases<br>· Not reported	Stage 1:<br>· DUKE A<br>· T1N0M0<br>· T2N0M0<br>Stage 2:<br>· DUKE B<br>· T3N0M0<br>· T4N0M0<br>Stage 3:<br>· DUKE C<br>· Regional<br>· T1-4N1M0<br>· T1-4N2M0<br>Stage 4:<br>· DUKE D<br>· Remote metastases<br>· T1-4N0-3M1<br>Stage X:<br>· Localized
Classification unique to DCR (1943–2003) *	· Localized<br>· Regional<br>· Remote metastases<br>· Not reported	
TNM classification (2004 to date) †	Consists of three categories: T indicates the extent of invasion of the primary tumor, N indicates spread to nearby lymph nodes, M indicates distant metastases.<br>T:<br>· T1<br>· T2<br>· T3<br>· T4	

	· Tx: Information on size of primary tumor not available<br>N:<br>· N0<br>· N1<br>· N2<br>· N3<br>· Nx: Information on lymph node status not available<br>M:<br>· M0: No distant metastases<br>· M1: Distant metastases identified<br>· Mx: Information on distant metastases not available	

Supplementary material 3

Figure S1. Kaplan Meier Curve showing the cumulative hazard of colon cancer incidence with time between the group with diverticulitis and the control group without diverticulitis. The curve only shows data on the cohort with no censoring from inclusion date and to six months after. The disease-free survival time starts six months after the inclusion date.

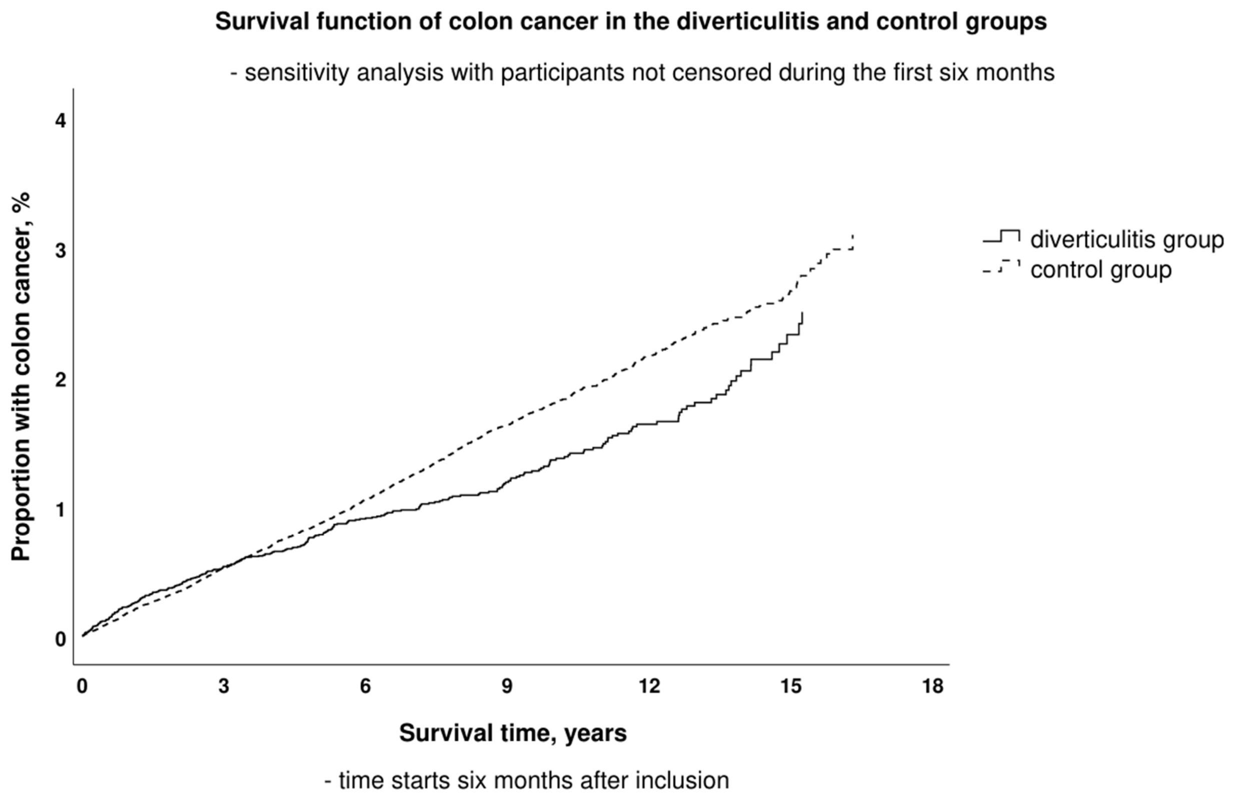

Supplement: Supplementary file 1 [file jcm-13-02503-s001.zip › jcm-2949309-supplementary.pdf]
